# Cystic pneumonia in a patient with AIDS

**DOI:** 10.1016/j.bjid.2023.102761

**Published:** 2023-03-25

**Authors:** Vincent Guiraud, Simon Bessis

**Affiliations:** AP-HP, Service de Maladies Infectieuses et Tropicales, CHU Bicêtre, Le Kremlin-Bicêtre, France

A 47-year-old man, HIV positive since 1991 and lost to follow-up for the last 10 years, presented in the emergency department for influenza-like syndrome, fever and cough. Pulmonary examination war unremarkable. Biological testing was noticeable for lymphocytopenia (lymphocyte 0.54 G/L) and uncontrolled HIV infection: high viral load (HIV1-RNA 5.81 log copies/mL), low CD4 T-cells level (CD3+CD4+ T-cells 7 mm^3^, CD3+CD8+ T-cells 83 mm^3^). Chest CT showed multiple bilateral cystic lesions, centrilobular micronodules and condensation, but lacked ground glass and mediastinal adenopathy (Fig. 1).

**Fig. 1:**
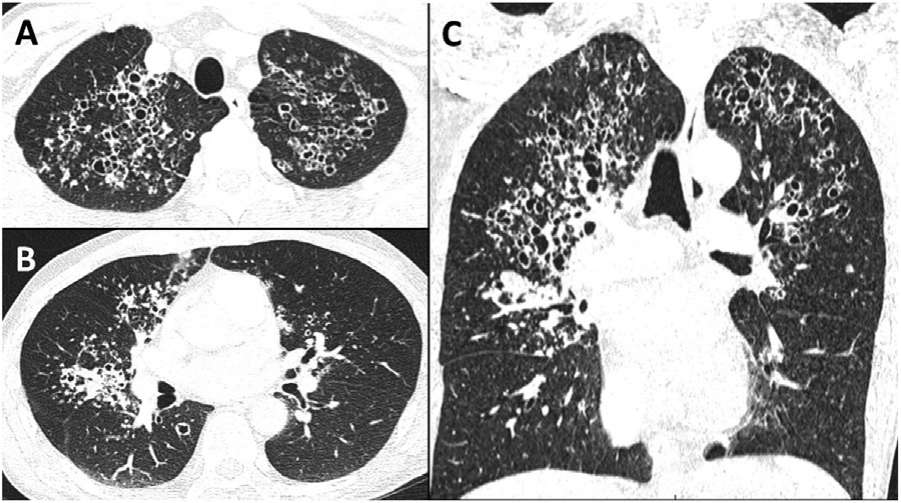
Axial CT image at the apex (A) and at the level below the tracheal bifurcation (B). Coronal CT image of the lungs (C).

Due to the advanced CD4 T-cell depletion infectious disease was most likely, with among them *Pneumocystis jirovecii*, past tuberculosis/active atypical mycobacterial infection and more infrequent paragonimiasis, echinococcosis and coccidioidomycosis.^1^ In this case, Cystic Pneumocystis Pneumonia (PCP) was diagnosed on sputum induced positive *P. jirovecii* quantitative PCR,^2^ while differential diagnosis were excluded through sputum and serum testing.

Previously described as one of the hallmark of PCP with diffuse ground glass opacities, cysts seems to become quite exceptional in non-AIDS patients.^3^ They have almost disappeared since the introduction of antiretroviral drugs. Interestingly, these cysts, unrelated to their size, tend to resolve within a year.^4^ It is noteworthy that this radiological pattern (lack or few ground glass and mostly apical cysts) is also characteristic of primary inhaled pentamidine prophylaxis failure.^5^ Finally, this case highlight to remember this simple saying: there is cyst in pneumocystis pneumonia.

## Funding information

The authors declare no specific funding.

## Data sharing statement

Patient gave written informed consent to share this image.

There is no identifying information in the image and text.

This article has been neither published nor submitted for publication elsewhere.

## Conflicts of interest

The authors declare no conflicts of interest.

## References

[1] Raoof S., Bondalapati P., Vydyula R. (2016). Cystic lung diseases: algorithmic approach. Chest.

[2] Sarasombath P.T., Thongpiya J., Chulanetra M. (2021). Quantitative PCR to discriminate between pneumocystis pneumonia and colonization in HIV and non-HIV immunocompromised patients. Front Microbiol.

[3] Christe A., Walti L., Charimo J. (2019). Imaging patterns of *Pneumocystis jirovecii* pneumonia in HIV-positive and renal transplant patients – a multicentre study. Swiss Med Wkly.

[4] Chow C., Templeton P.A., White C.S. (1993). Lung cysts associated with *Pneumocystis carinii* pneumonia: radiographic characteristics, natural history, and complications. AJR Am J Roentgenol.

[5] Edelstein H., McCabe R.E. (1990). Atypical presentations of *Pneumocystis carinii* pneumonia in patients receiving inhaled pentamidine prophylaxis. Chest.

